# Economic burden of dengue in Indonesia

**DOI:** 10.1371/journal.pntd.0007038

**Published:** 2019-01-10

**Authors:** Mardiati Nadjib, Ery Setiawan, Septiara Putri, Joshua Nealon, Sophie Beucher, Sri Rezeki Hadinegoro, Vetty Yulianty Permanasari, Kurnia Sari, Tri Yunis Miko Wahyono, Erna Kristin, Dewa Nyoman Wirawan, Hasbullah Thabrany

**Affiliations:** 1 Health Policy and Administration Department, Faculty of Public Health, Universitas Indonesia, Depok, Indonesia; 2 Centre for Health Economics and Policy Studies, Faculty of Public Health, Universitas Indonesia, Depok, Indonesia; 3 Sanofi Pasteur, Asia & JPAC region, Singapore; 4 Faculty of Medicine Universitas Indonesia, Cipto Mangunkusumo Hospital, Jakarta, Indonesia; 5 Department of Epidemiology, Faculty of Public Health, Universitas Indonesia, Depok, Indonesia; 6 Department of Pharmacology & Therapy, Faculty of Medicine, Universitas Gadjah Mada, Yogyakarta, Indonesia; 7 Faculty of Medicine, Universitas Udayana, Denpasar, Bali; Brandeis University, UNITED STATES

## Abstract

**Background:**

Dengue is associated with significant economic expenditure and it is estimated that the Asia Pacific region accounts for >50% of the global cost. Indonesia has one of the world’s highest dengue burdens; *Aedes aegypti* and *Aedes albopictus* are the primary and secondary vectors. In the absence of local data on disease cost, this study estimated the annual economic burden during 2015 of both hospitalized and ambulatory dengue cases in Indonesia.

**Methods:**

Total 2015 dengue costs were calculated using both prospective and retrospective methods using data from public and private hospitals and health centres in three provinces: Yogyakarta, Bali and Jakarta. Direct costs were extracted from billing systems and claims; a patient survey captured indirect and out-of-pocket costs at discharge and 2 weeks later. Adjustments across sites based on similar clinical practices and healthcare landscapes were performed to fill gaps in cost estimates. The national burden of dengue was extrapolated from provincial data using data from the three sites and applying an empirically-derived epidemiological expansion factor.

**Results:**

Total direct and indirect costs per dengue case assessed at Yogyakarta, Bali and Jakarta were US$791, US$1,241 and US$1,250, respectively. Total 2015 economic burden of dengue in Indonesia was estimated at US$381.15 million which comprised US$355.2 million for hospitalized and US$26.2 million for ambulatory care cases.

**Conclusion:**

Dengue imposes a substantial economic burden for Indonesian public payers and society. Complemented with an appropriate weighting method and by accounting for local specificities and practices, these data may support national level public health decision making for prevention/control of dengue in public health priority lists.

## Author summary

Dengue, an infection transmitted by mosquitos, is a public health concern particularly in tropical/subtropical areas and the Asia Pacific region where it is associated with a significant cost to society. Indonesia has one of the world’s highest dengue burdens but Indonesia-specific data on cost are lacking. To estimate the annual economic burden of dengue in Indonesia, this study collected data from public/private hospitals and health centres in three provinces (Yogyakarta, Bali and Jakarta) during 2015. We estimated cost of illness using the societal perspective: calculations of costs included those that were directly paid by the healthcare system, as well as costs incurred by the patients (or their family/care givers) and their lost productivity. The costs from the three provinces were then used as the basis for extrapolating cost of illness in Indonesia. The authors confirmed that dengue imposed a substantial economic burden for Indonesian public payers and society. Based on 2015 data, the authors estimated total economic burden of dengue in Indonesia at US$381.15 million. Of this, US$355.2 million related to patients treated in hospitals and US$26.2 million was for patients treated in health centres. Establishing a better understanding of the burden of dengue in Indonesia will help to guide public health decision-making at a national level and support prevention and control initiatives for this disease.

## Introduction

Dengue is an arboviral infection transmitted between humans by *Aedes* mosquitoes. Globally, dengue is a major public health concern that has rapidly spread across the tropics and subtropics.[1, 2] Between 1990 and 2013 the estimated number of global dengue cases doubled every decade,[3] and up to 3.9 billion people remain at risk in endemic countries.[4] Recent global modelling studies estimate between 55–100 million dengue cases occur annually; and estimate an increasing dengue mortality reaching over 38,000 deaths in 2016.[3, 5, 6] Of the global population at risk, more than 70%–or about 1.8 billion people–live in the Asia-Pacific region and as such, Asians contribute the most to the overall burden of dengue.[1] In addition, the incidence of the severe forms of disease is higher in Asia-Pacific compared with other endemic regions perhaps for reasons of genetic susceptibility, but more likely because secondary infection is more common, due to the higher levels of endemicity and that all four dengue virus serotypes continually co-circulate.[7–10]

In Indonesia, *Ae*. *aegypti* and *Ae*. *albopictus* are the primary and secondary vectors for transmission, respectively. The average number of annual dengue cases reported to health authorities in Indonesia was more than 129,000 for the period between 2004 and 2010, the second highest incidence rate in the world after Brazil.[1] Reporting of dengue in Indonesia is acknowledged to be incomplete and reporting procedures vary widely among the provinces.[11] A 2013 cartographical modelling study estimated that approximately 7.6 million dengue infections may have occurred in in Indonesia in 2010, the majority of which went unreported.[5] The disease typically is most common in urban areas, however, rural areas are increasingly affected.[7] Furthermore, the traditionally cyclical epidemic outbreaks of dengue appear to have become more erratic in recent decades.[9]

The costs associated with dengue illness are substantial, in 2012 the WHO ranked dengue as the most important mosquito-borne viral disease across the globe, noting that outbreaks “exert a huge burden on populations, health systems and economies in most tropical countries of the world”.[1] Recognizing the substantial impacts in endemic regions, several economic burden studies have been conducted in various regions of the Americas,[12–18] and several countries in Asia and South Asia including Thailand,[19] Malaysia,[20, 21] India,[22] Singapore,[23] Cambodia [24] and the Philippines.[25] These studies confirmed the considerable direct and indirect impact of dengue on individuals, families and communities.

In Indonesia, some initial insights could be derived from the study by Shepard and colleagues, who estimated the annual economic burden of dengue in 12 countries of Southeast Asia at US$950 million; for Indonesia the annual cost over the period 2001–2010 was US$323 million.[26] A subsequent estimate based on revised global dengue incidence estimates and extrapolations of costs from scientific literature estimated the costs in Indonesia in 2016 to have been US$2 billion.[27] Another study by Stahl and colleagues estimated the cost of dengue outbreaks by conducting a literature review and case studies in four countries including Indonesia.[28] The estimated costs of an Indonesia dengue outbreak in 2011 were US$6.75 million (adjusted to 2012 US$). However, these studies did not collect local empirical cost data and instead relied on estimates derived from a literature review on unit costs for inpatient and outpatient care and used extrapolations of proportionality of costs from other nearby countries.[26–28] One study conducted in Surabaya, Indonesia in 2007 examined treatment costs at the hospital level and estimated inpatient costs per episode related to dengue were in the range of 1–2 million Indonesian Rupiah (IDR) or approximately US$106–212. However, the scope of this study was limited to that single area and did not provide country-wide estimates for total healthcare costs.[29]

We are not aware of a study which has collected comprehensive primary data on the economic burden of dengue in Indonesia. Such studies are needed to inform policy making, provide information to support healthcare resource allocation including prioritizing research and disease prevention and control measures, as well as promote public awareness.[30] Due to the country’s economic, geographic and sociological heterogeneity, the best way to represent national level burden and expenditure would be to use data from multiple sites and treatment facilities, taking a broad economic perspective. The aim of this study was to estimate the economic burden–including direct and indirect costs–associated with hospitalized and ambulatory dengue cases in Indonesia, first by determining costs at the facility level across three provinces, then extrapolating these using local epidemiological data to make the first, empirically-derived national economic burden of dengue estimates for Indonesia.

## Methods

This study used a combination of retrospective and prospective methods and multiple data sources to estimate the direct and indirect costs of dengue in Indonesia as of 2015.

### Ethical considerations

The ethics Committee of the Faculty of Public Health at Universitas Indonesia approved this study. Ethical approval for data collection at public hospitals and health centres was received from the local authorities (Dinas Kesehatan or District Health Office). Interview participants or their parents/guardians signed informed consent (signed assent forms were also required for those aged 8–18 years) before study entry.

### Study sites

In Indonesia, tertiary healthcare facilities are divided into type A facilities, which provide the full spectrum of specialist medical services and type B facilities, where specialist services are limited. Both types provide basic and supportive care to both in- and out-patients. Of the 34 provinces in Indonesia, three were selected to represent low- (Yogyakarta), medium- (Bali) and high-income (Jakarta); from these three a total of nine facilities were selected for inclusion in the study. Public and private healthcare facilities were selected according to their research experience; and to provide a range of dengue and cost perspectives, including those treating inpatients and outpatients. Four facilities were selected in Jakarta: RSUPN Cipto Mangunkusumo (public type A hospital), RSUD Pasar Rebo (public type B hospital), RS Pelni (private hospital) and Tambora (Puskesmas [a sub-district level public health centre]); three facilities in Yogyakarta: RSUD Wirosaban (public type B hospital), RS Bethesda (private hospital), Puskesmas kota Yogyakarta (Puskesmas); two facilities in Bali: Sanglah Hospital (public hospital) and Puskesmas VI Denpasar (Puskesmas).

### Sampling strategy

Patient records were randomly selected from a list of all age-stratified dengue diagnoses, maintained in facility diagnosis log books, in the 12 months preceding the beginning of the study. We planned to assess 50 inpatient and 50 outpatient records from each hospital (total sample from six hospitals = 600); and 50 outpatient records from each Puskesmas (total sample from three sites = 150). It was expected that the sample would comprise an equal number of children (≤18 years old) and adults (≥ 19 years old) due to the approximately equal distribution of dengue cases occurring in these categories. Additionally, we intended to interview 30 inpatients and 30 outpatients or their respective parents/guardians at each hospital (total sample from six hospitals = 360) and 30 outpatients or their parents/guardians from each Puskesmas (total sample from three sites = 90). Sample sizes were chosen to be operationally feasible and sufficiently large that analysis methods based on the normal distribution may be used for the analysis.

### Sources of data–direct medical costs from patient records

Direct medical costs were retrospectively assessed through review of medical records and billing/charges made to patients who received treatment at selected hospitals or Puskesmas (sub-district level health centres) in the 12 months prior to the beginning of the study (April 1^st^ 2014 until March 31^st^ 2015) with a diagnosis of dengue or dengue haemorrhagic fever (with ICD 10 code A90 and A91).

### Sources of data–direct non-medical and indirect medical costs from interviews

Direct non-medical costs and indirect costs were assessed from data collected during face-to-face interviews with patients or their parents/guardians at selected hospitals or Puskesmas. Patients with clinically diagnosed/laboratory confirmed dengue or those with evidence of fever >38˚C for >1 day, plus symptoms compatible with dengue fever were recruited to participate in two interviews. The first was a face-to-face interview with patients or their parents/guardians using a questionnaire and conducted at the health facility at discharge/during an ambulatory visit. The second interview was conducted by telephone two weeks later to determine subsequent costs of treatment and any absenteeism from work/school. Direct non-medical costs were defined to include all expenses incurred due to the treatment, such as meals, transport, accommodation for care givers, etc. The interviews documented: the use of medical services; missed schooling; lost work productivity; out-of-pocket spending (e.g. transportation, meals, hotel/house rental, etc) and income lost due to the episode of illness. In the event that participants chose not to disclose their income and in the absence of reliable data on average wages including in the informal economy, we applied the standard minimum wages as a proxy, which are regulated in Indonesia and differ by province. Lost productivity was not calculated for children; rather, for each affected school child lost productivity was calculated for the caregiver (as a result of leaving work to care for the child).

### Cost of dengue cases

Costs were expressed in US dollars (as of 2016 with a conversion rate: US$1 = IDR13,000). For those regions where a particular type of facility was not included in the study, gaps in the data were filled via weighted adjustment from neighbouring sites. For example, private outpatient costs were captured by recording treatment bills paid by the patient in Jakarta. Because private outpatient facilities were absent in Yogyakarta, these costs were estimated by adjusting Jakarta values weighted according to outpatient public costs for Jakarta and Yogyakarta. In Bali, private outpatient and inpatient costs were estimated based on the ratio observed in Yogyakarta.

### Extrapolation of the dengue cost burden to the national level

Passive reporting of dengue in Indonesia is mandatory within 72 hours of diagnosis according to SEARO-WHO dengue diagnosis guidelines 2011.[31] Notification follows diagnosis by clinical and/or laboratory confirmation (by detection of NS1 antigen and/or IgM/IgG). Cases are reported to provincial health offices and pooled at the provincial and national levels by the Directorate General of CDC.[32]

Costs at the national level were estimated by multiplying cost per case (outpatient/inpatient) by an estimate of the number of cases occurring in Indonesia in 2015. National burden estimates were generated using a) provincial-level surveillance data from each of the 34 provinces; b) estimates of hospitalization rate derived from an expert consensus technique in Indonesia;[11] and c) a study which observed a magnitude of dengue under-reporting of 11.5-fold in the placebo group of a dengue vaccine clinical trial in Jakarta, Bandung and Denpasar, Bali.[33]

The expert panel that gave rise to the estimates of hospitalization rate has been described previously;[11] briefly, it comprised a group of Indonesian dengue experts (clinicians, hospital managers, epidemiologists and Ministry of Health officials) who reviewed existing data sources and made iterative estimates of epidemiological parameters by which full burden estimates could be made. These were balanced against published analyses.[3, 5, 33–37] The panel concluded that 60% of dengue cases in Indonesia were hospitalized; a figure which, when combined with an estimated reported hospitalization rate and under-reporting factor of 11.5, generated the final expansion factor for hospitalized patients (EFH; 7.65) and expansion factor for ambulatory patients (EFA; 45.90) used for calculation of the cost-of-illness. The numbers of ambulatory and hospitalized dengue cases for each province in Indonesia during 2015 were estimated by multiplying these expansion factors by the numbers of reported cases in each province.

To calculate the economic burden of dengue nationally the three sites in our study: Jakarta, Yogyakarta and Bali, were used as references for other provinces arranged into three groups according to their fiscal capacity index (FCI). Yogyakarta was the reference for low FCI province (FCI <0.5), Bali for middle FCI (0.5–2.0) and Jakarta for high FCI (>2.0) provinces. Unit outpatient and inpatient costs of each province were proportionally weighted by the consumer price index (CPI) or Indeks Harga Konsumen (IHK) using Jakarta, Bali and Yogyakarta dengue unit costs as the baseline. By multiplying the number of ambulatory/hospitalized cases by the unit cost estimates for each province, the total economic burden in each province was calculated.[11]

### Sensitivity analyses

To assess the uncertainty surrounding estimated overall dengue burden,[38] deterministic sensitivity analyses were performed to examine the effect of parameters’ variations i.e. costs in each setting (inpatient, outpatient, by region) and expansion factor. Each parameter was manually varied by an arbitrary value of ±10% to examine the impact on the total economic burden. Calculations were performed using Microsoft Office Excel 2010.

## Results

### Data collection, recruitment and timelines

A total of 615 patient records were reviewed for the retrospective, direct medical cost calculation (262 in Jakarta, 251 in Yogyakarta and 102 in Bali) during the period from the 15^th^ of June to the 31^st^ of July 2015. The regional distribution of patients included, by province, inpatient/outpatient and dengue classification is shown in Table 1. A total of 199 patients were involved in the prospective phase of the study (94, 43 and 62 from each site); data were collected from interviews during the period from the 3^rd^ of August to the 15^th^ of September 2015. Combining both retrospective and prospective elements, the study sample was 68% of the enrolment target.

**Table 1:** Distribution of patient records used in the retrospective analyses, by province, type of service and severity of dengue during the period from 15^th^ of June to 31^st^ of July 2015.

Province	Type of services	Dengue fever	Dengue haemorrhagic fever	Dengue shock syndrome	Total
Yogyakarta	Outpatient	33	15		48
	Inpatient	3	48	3	54
Bali	Outpatient	109	8		117
	Inpatient	50	82	2	134
Jakarta	Outpatient	62	56		118
	Inpatient	43	94	7	144
**Total**		300	303	12	615

### Direct and indirect medical cost for inpatient and outpatient care of dengue cases in Jakarta, Yogyakarta and Bali

The total costs (combined direct and indirect costs) per patient episode for outpatient cases were, US$103, US$252 and US$179 for Yogyakarta, Bali and Jakarta, respectively. For inpatients these costs were US$689, US$989 and US$1071 respectively. With the exception of inpatient costs in Jakarta, direct medical costs were higher from private hospitals compared with public facilities. Table 2 describes cost of illness results per episode for each site. Direct medical cost were the largest proportion of costs for inpatient care, while indirect costs were the largest proportion of costs for outpatient care. Outpatient costs in Jakarta were slightly lower than in Bali. Overall, the mean length of hospital stay was 3.9 days. By region, it was 4.4 days in Yogyakarta, 3.5 for Bali and 3.8 days in Jakarta.

**Table 2:** Cost of illness per patient per episode (in US$) by component and site in Yogyakarta, Bali and Jakarta.

Province	Type of services	Health facility	Direct costs: medical (US$)	Direct cost: non-medical (US$)	Indirect cost (US$)	Total cost (US$)
Yogyakarta	Outpatient	Puskesmas	3.32	21.15	8.02	32.49
		Public hospital	7.33	10.00	8.02	25.35
		Private hospital	26.66	10.00	8.02	44.68
	Inpatient	Public hospital	222.89	18.31	46.16	287.35
		Private hospital	334.30	24.19	43.06	401.55
Bali	Outpatient	Puskesmas	16.73	15.14	30.64	62.51
		Public hospital	20.08	21.31	49.54	90.92
		Private hospital	28.13	21.31	49.54	98.98
	Inpatient	Public hospital	229.55	76.89	122.31	428.75
		Private hospital	344.30	101.61	114.11	560.01
Jakarta	Outpatient	Puskesmas	14.53	6.64	13.77	34.94
		Public hospital	23.41	10.47	30.32	64.19
		Private hospital	32.80	17.64	29.08	79.52
	Inpatient	Public hospital	407.07	84.33	153.79	645.19
		Private hospital	248.61	73.24	104.12	425.97

### Total economic burden due to dengue in the provinces of Indonesia

The results of the extrapolated regional costs (by CPI and FCI) are shown in Table 3. Jakarta was the province with the highest dengue-related cost, followed by Yogyakarta, West Java and West Kalimantan.

**Table 3:** Extrapolated total economic burden (in US$) of dengue in Indonesian provinces by income-category (Region 1: Low income context, Region 2: Medium income context, Region 3: High-income context). Number of cases estimated by extrapolation from cases reported from passive surveillance in 2015 using expansion factors.

	Actual inpatient cases	Total inpatient cost (US$)	Actual outpatient cases	Total outpatient cost (US$)	Total economic burden (US$)
REGION 1					
Yogyakarta	139,556	48,070,196	92,635	3,165,523	51,235,719
Bengkulu	4,317	1,535,502	2,866	101,116	1,636,618
Banten	34,331	12,152,270	22,788	800,252	12,952,522
West Sumatra	26,376	9,233,246	17,508	608,029	9,841,274
North Sumatra	36,549	12,738,624	24,260	838,865	13,577,489
Jambi	23,534	8,146,013	15,622	536,432	8,682,445
West Java	113,638	39,173,980	75,431	2,579,688	41,753,669
Special Region of Aceh	10,457	3,521,684	6,941	231,910	3,753,594
South Sumatra	6,951	2,340,043	4,614	154,097	2,494,140
North Kalimantan	4,484	1,628,835	2,976	107,262	1,736,097
West Kalimantan	74,179	25,967,594	49,238	1,710,020	27,677,614
East Java	20,866	7,209,095	13,851	474,734	7,683,829
Central Java	23,701	8,186,530	15,732	539,100	8,725,631
West Nusa Tenggara	10,395	3,637,047	6,900	239,507	3,876,554
Maluku	3,368	1,177,939	2,236	77,570	1,255,509
East Nusa Tenggara	1,601	551,161	1,063	36,295	587,456
Southeast Sulawesi	10,887	3,732,207	7,227	245,774	3,977,980
North Sulawesi	10,409	3,546,774	6,909	233,562	3,780,337
West Sulawesi	4,983	1,696,603	3,307	111,725	1,808,328
South Sulawesi	5,059	1,721,320	3,358	113,353	1,834,673
Gorontalo	25,419	8,546,321	16,873	562,793	9,109,114
Central Sulawesi	44,754	15,659,385	29,707	1,031,203	16,690,588
Special Region of Papua	457	60,387	304	10,562	170,949
Lampung	12,439	4,467,725	8,257	294,209	4,761,934
**Total region 1**	648,710	224,700,481	430,603	14,803,581	239,604,063
REGION 2					
Bali	27,540	13,615,216	18,280	1,538,048	15,153,264
Special Region of West Papua	825	396,267	547	44,764	441,031
Riau	22,599	11,207,903	15,001	1,266,105	12,474,008
Bangka–Belitung Islands	21,795	11,038,270	14,467	1,246,942	12,285,212
Riau Islands	9,383	4,609,852	6,228	520,754	5,130,605
Central Kalimantan	8,676	4,246,122	5,759	479,665	4,725,787
South Kalimantan	3,507	1,704,769	2,328	192,580	1,897,349
North Maluku	541	272,137	359	30,742	302,879
**Total region 2**	94,866	47,090,536	62,969	5,319,600	52,410,135
REGION 3					
Jakarta	147,172	78,822,769	97,690	5,817,201	84,639,971
East Kalimantan	7,727	4,188,784	5,129	309,137	4,497,920
**Total region 3**	154,899	83,011,553	102,819	6,126,338	89,137,891
**NATIONAL TOTAL**	**898,475**	**354,802,570**	**596,391**	**26,249,519**	**381,152,089**

### Total economic burden due to dengue in Indonesia

The annual total cost of dengue-related illness in Indonesia was estimated at US$381.5 million, with US$354,802,570 for hospitalised and US$26,249,519 for ambulatory cases (Table 3). Considering the total number of inpatient cases and costs, the average cost per dengue patient was lowest in region 1 ($346.38) and highest in region 3 (US$535.91). Similarly, average cost per outpatient was lowest in region 1 (US$34.38) and highest in region 2 (US$84.48).

### Sensitivity analysis

**Fig 1:**
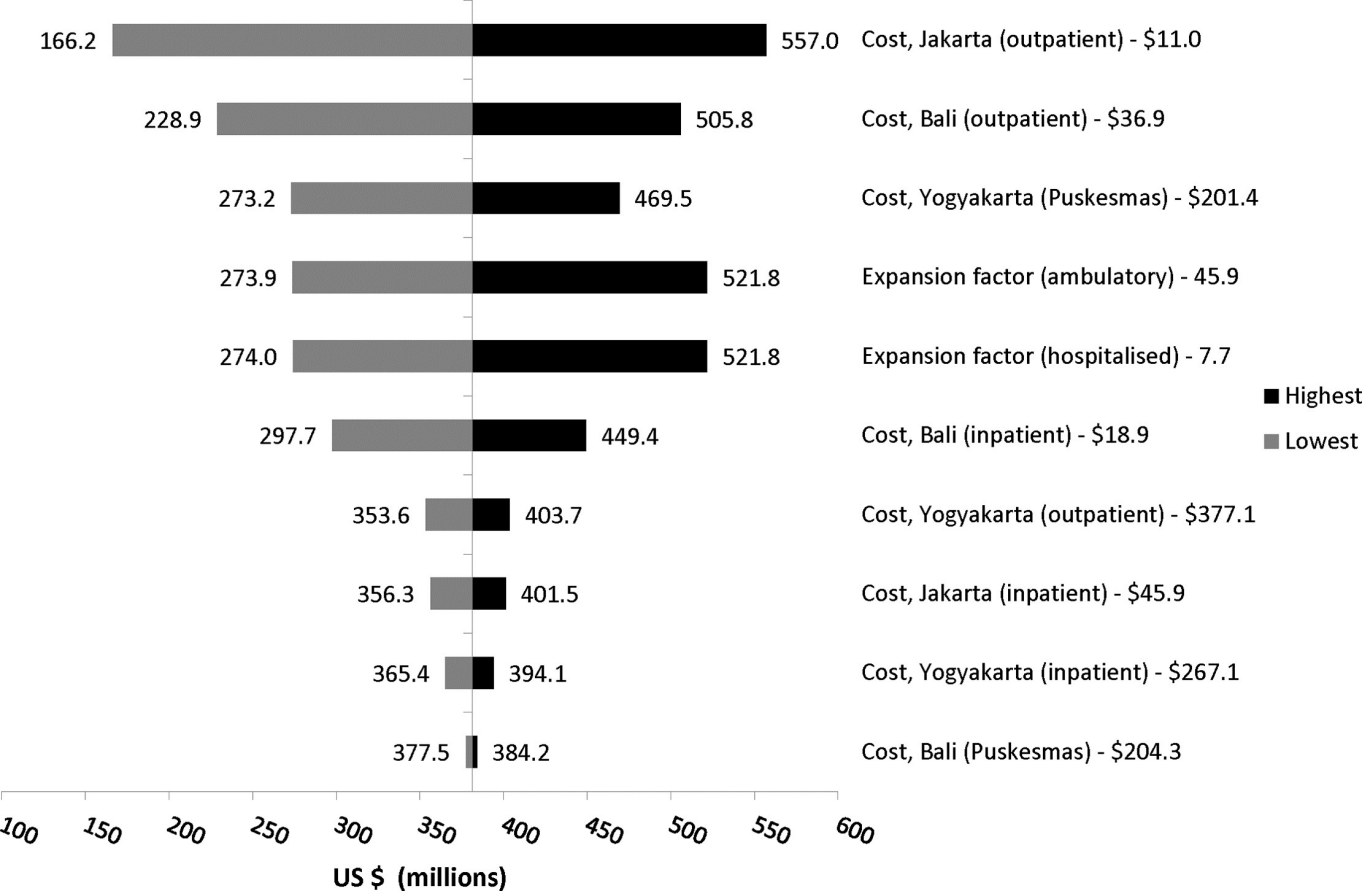
Tornado diagram for the deterministic sensitivity analysis of variability of the Indonesian national-level, annual cost of dengue illness in US$ million. Black represents the lowest value, grey represents the largest value. Parameters were varied by ±10% as a subjective scenario and the base case was US$381.15 million. The point estimate for each parameter is included in the label for each bar.

Results from the sensitivity analyses are presented in the Tornado diagram (Fig 1), which represents baseline value (per US$ million). The parameters included in sensitivity analysis were the costs of outpatient and inpatient treatment by province; and EFA and EFH. Variation in any of these parameters resulted in overall economic burden varying from US$166–557 million. The greatest variation in the final estimate followed variation in outpatient costs in Jakarta; followed by costs in outpatient facilities in type A clinics, and in Bali.

## Discussion

We estimated the average annual economic burden of dengue-related illness in Indonesia in 2015 to be US$381.5 million with more than 90% of this cost associated with hospitalized care. Jakarta was the province associated with the greatest cost, which is a function of the greater population and the higher average costs of treating hospitalized dengue episodes. In Jakarta, inpatient, direct medical costs were higher from public facilities than in private hospitals. This was thought to result from the fact that the public study sites included Ciptomangunkusumo Hospital which is a type A public hospital, a top referral hospital in Indonesia and therefore responsible for treating the most severe cases requiring intensive, expensive, specialist care. Sensitivity analyses identified uncertainty around outpatient cost in Jakarta as the variable with the largest impact on the overall economic burden, due to the relatively higher cost of episodes in Jakarta, and their frequency. Notably, the overall estimates are directly influenced by the expansion factors used to estimate the number of cases. These numbers were derived from high-quality epidemiological studies in tandem with local expert opinion. But studies have shown reporting completeness can be affected by changes in disease severity, level of epidemic activity and other external factors, which could limit the generalizability of these numbers at different time points. 2015 was a fairly “typical” year in Indonesia, with the number of cases being close to the average from 2010–2016.[39, 40] Future analyses will hopefully allow for a more refined understanding of the level of dengue reporting in Indonesia.

Our estimate of the cost per episode in type B hospital was~US$150 (~IDR 2 million), which is consistent with a previous Indonesian estimate from East Java of IDR1–2 million published in 2008.[29] Our unit cost estimates are also similar to those reported in the regional analyses of Shepard in 2013 and 2016.[26, 27] Our study found that dengue is associated with considerable economic burden, which is in agreement with other studies conducted in Asian countries, especially those in Thailand and the Philippines. In Thailand, Philippines and Malaysia, total economic burdens were estimated at US$486 million (in 2005 costs),[19] US$345 million (in 2012 costs),[25] US$102.25 million (in 2009 costs),[20, 21] respectively. However, estimates in the much smaller (Singapore) and larger (India) countries were considerably higher than our estimate at more than US$1 billion in each country.[23, 41]

With regard to existing national level burden estimates for Indonesia, our results are similar to those published by Shepard and colleagues in 2013 who concluded that the annual economic burden of dengue for Indonesia was US$323 million.[26] This was slightly lower than our 2015 estimate, caused by an increasing disease burden; and slightly higher outpatient unit costs. However, this group refined their estimates in a 2016 [27] publication using a different method of epidemiological burden estimation and concluded that the dengue burden in Indonesia was US$2 billion.[27] Costs in our study were calculated from primary data sources and clinically diagnosed dengue, including medical record review and patient interview. Unit costs were broadly similar to those estimated by Shepard and colleagues and the variation is predominantly driven by different epidemiological estimates: Shepard and colleagues’ estimated >11 million annual dengue cases, while we assumed ~640,000. Such variation in dengue burden estimates are difficult to reconcile; the paper by Shepard and colleagues applied regression methods from the Global Disease Burden group; in contrast we used local surveillance data combined with expert opinion and empirical under-reporting calculation. Much of this variation likely stems from case definitions, particularly those around mild cases of dengue whose clinical and economic significance is very difficult to calculate with confidence, and whose full economic impacts are very difficult to measure. In addition, the Shepard 2016 study included estimates for non-medical cases (i.e. patients that did not seek professional medical advice but may have had laboratory testing or purchased therapeutic products outside the professional healthcare system), which we did not include in this analysis.

The strength of this study is that it is based on empirical, patient-specific data for medical care and out-patient costs in Indonesia. Furthermore, it considered both public and private hospitals and included costs derived from different treatment settings and economic backgrounds. To address limitations in the available passive surveillance data, expansion factors were used to fully describe the number of dengue cases and expert opinion employed to desegregate data into outpatient and inpatient cases. We consider this approach, underpinned by gold-standard epidemiological clinical trial data with local expert opinion to stratify cases by severity, is likely a realistic representation of the health-seeking dengue case population in Indonesia. The costs captured from the three reference provinces (Jakarta, Bali and Yogyakarta) were extrapolated to other regions based on weighted average costs linked to the consumer price index to ensure relevant estimates from other regions. Other variables such as type of hospitals (private/public, type A or B) were also taken into account in the extrapolation to get a mixed representation of healthcare setting throughout the country.

We acknowledge several limitations to our study, mostly due to the patients’ clinical pathway i.e. most patients generally received outpatient services at type B hospitals, hence had an impact on type A sample size; also the number of ambulatory patients was generally lower than expected (potentially due to the local regulation at Jakarta and Yogyakarta whereby laboratory-confirmed dengue patients were referred directly to hospital). Furthermore, we did not enroll as many patients as planned and were only able to achieve 68% of the target enrolment. The primary reason for lower-than-expected enrolment was the relatively small number of dengue cases occurring in 2015, especially in Yogyakarta in which enrolment was especially challenging. Outpatient recruitment was additionally complicated by local clinical practice guidelines which advise that all dengue cases should be hospitalized. There is uncertainty in income loss calculations due to illness because most patients or their parents/guardians did not disclose their actual income during the interviews; so the national minimum wage was used as proxy. Some studies also included ‘outside hospital costs’, such as vector control activities, in the overall cost estimates, but this was beyond the scope/focus of our study. Lastly, our estimates are based on data from one year (2015), corresponding to the period over which primary data were collected. As a result, the estimates are subject to vary with epidemic activity.

## Conclusion

The total direct costs of dengue illness in Indonesia were estimated at US$381.15 million. Our analysis provides results that are relevant to public health policymakers in Indonesia, helping to strengthen local knowledge and informing decision-making regarding the prevention and control of dengue in public health priority lists. These results can also be used in health economic studies of novel dengue prevention and control technologies or vaccine programs.

## Supporting information

S1 Table STROBE statement—checklist of items that should be included in reports of cross-sectional studies. (DOC)
